# Acute spinal cord compression in the setting of chronic extramedullary hematopoiesis of the thoracic spine

**DOI:** 10.1016/j.xnsj.2023.100260

**Published:** 2023-08-03

**Authors:** Ty Agaisse, Cameron Thomson, Mariah Balmaceno-Criss, Leland McCluskey, Bassel G. Diebo, Eren Kuris, Alan H. Daniels

**Affiliations:** Department of Orthopedic Surgery, Warren Alpert Medical School, Brown University, 1 Kettle Point Ave, East Providence, RI 02914, United States

**Keywords:** Extramedullary hematopoiesis, Thalassemia, Intravertebral vacuum phenomenon, Compression fracture, Spinal cord injury

## Abstract

**Background:**

Though rare, pathologic extramedullary hematopoiesis (EMH) can occur in response to myeloproliferative disorders and may present as paravertebral masses.

**Case Description:**

We describe a 63-year-old female with unspecified thalassemia, hemochromatosis, and known asymptomatic extramedullary hematopoiesis of the thoracic spine who acutely developed severe spinal cord compression and a T9 vacuum phenomenon fracture 7 months after her initial diagnosis.

**Outcome:**

The patient was treated with urgent decompression and T9 kyphoplasty, which resulted in complete resolution of her neurological deficits.

**Conclusions:**

The timeline of symptomatology in the case suggests that asymptomatic patients with T-spine extramedullary hematopoiesis can develop progressive neurologic deterioration and atraumatic compression fractures culminating in acute spinal cord injury. While it may be appropriate to treat asymptomatic patients conservatively, surgical decompression must always remain a consideration.

## Introduction

In extramedullary hematopoiesis (EMH), hematopoietic stem cells expand and differentiate, producing blood cells outside the bone marrow [1]. While this can be a physiologic response in times of increased oxygen demands, pathological EMH can occur in several conditions, such as hematopoietic disorders, insufficient hematopoietic compensation, anemia, infection, tumors, and metabolic stress [1]. A prominent cause of EMH is thalassemia, a congenital deficiency of hemoglobin production resulting in chronic anemia [2].

EMH is most commonly found in the liver and spleen but can also occur in the lung, kidney, and vertebral column [3]. Vertebral EMH is reported in 11% to 15% of all EMH cases, most often occurring in the thoracic spine [2]. Due to the rarity of EMH, literature describing vertebral EMH is limited to case reports and series. Vertebral EMH is frequently diagnosed incidentally, as many patients are asymptomatic [2]. However, given the limited epidural space in the thoracic spine, the mass effect from EMH can lead to neurological deficits ranging from mild radiculopathy to severe spinal cord compression [4].

Here, we present a patient with known thoracic spine EMH and multiple thoracic and lumbar spine compression fractures, being treated conservatively, who developed acute onset of severe spinal cord compression and a T9 fracture with vacuum phenomenon. The patient subsequently underwent urgent multilevel decompression and T9 balloon kyphoplasty resulting in full neurologic recovery. We obtained informed consent to share the case.

## Case report

**Fig. 1:**
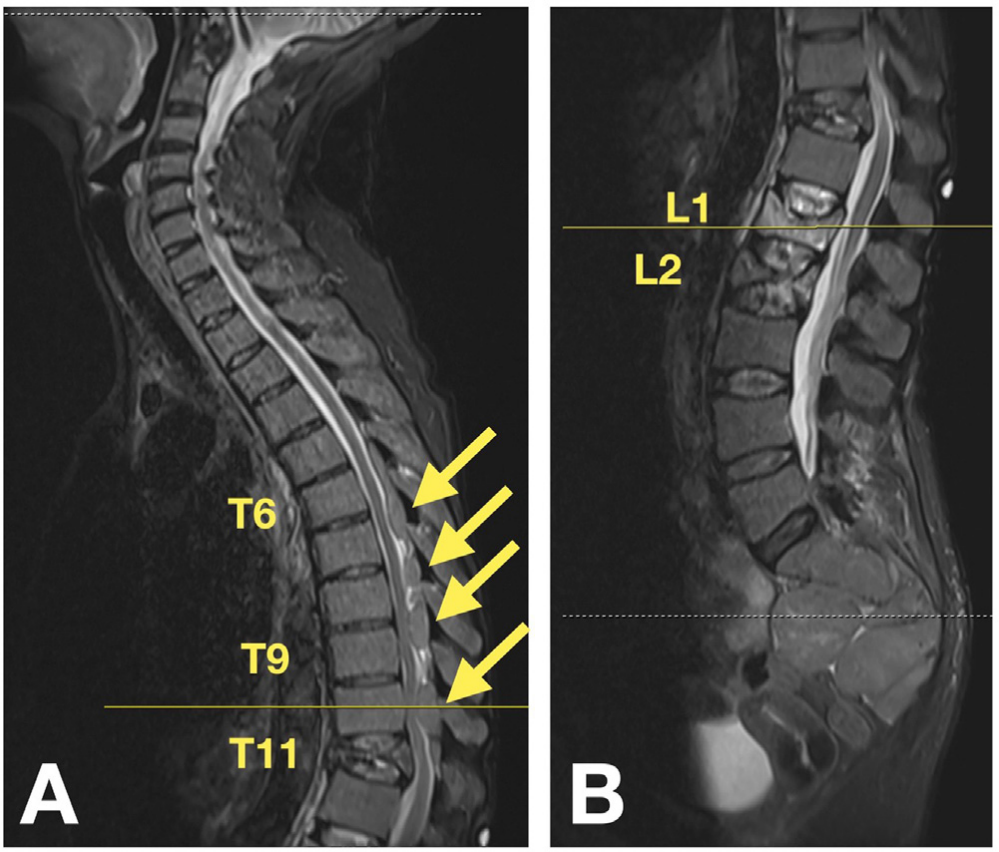
Initial thoracic (A) and lumbar (B) MRI demonstrating compression fractures at T11, L1, and L2 and the dorsal extradural masses from T6 to T10.

A 63-year-old female with a past medical history of unspecified thalassemia and hemochromatosis presented to our emergency department with worsening chronic back pain and an acute inability to ambulate with no preceding trauma. Seven months before arrival, the patient had suffered a mechanical fall and presented to an outside hospital with a chief complaint of back pain. At that time, magnetic resonance imaging (MRI) revealed acute compression fractures at T11, L1, and L2 and multiple dorsal extradural masses at the levels T6 to T10 (Fig. 1). The patient followed up with an outside spine surgeon, who recommended referral to a pain clinic for management of her back pain, as opposed to surgical intervention, given the lack of neurological deficits.

**Fig. 2:**
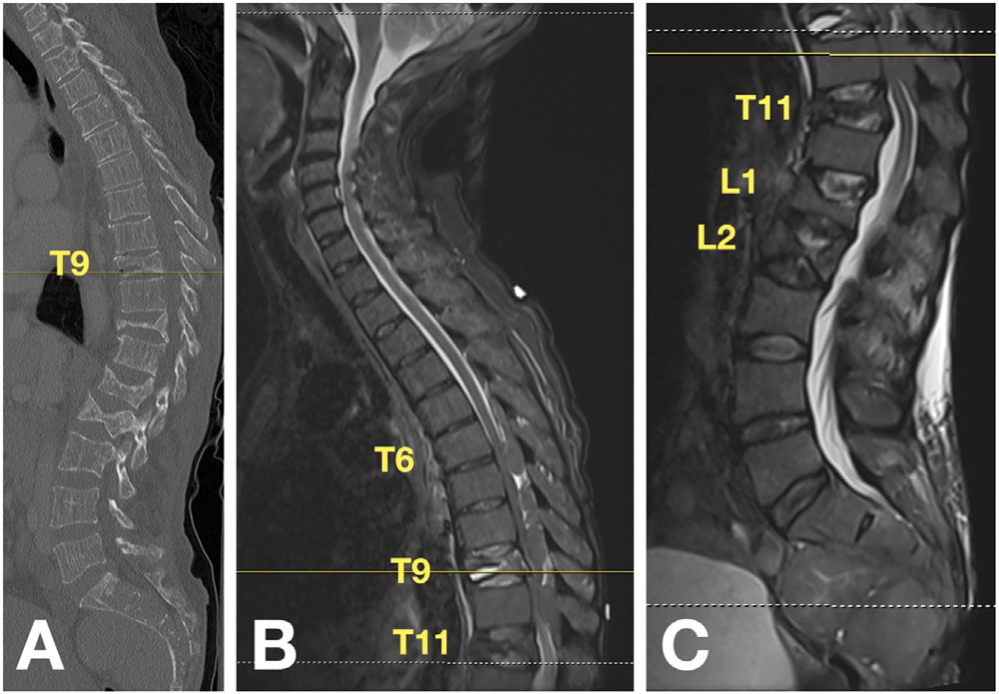
Seven months after initial presentation, CT (A) demonstrating acute T9 compression fracture with vacuum phenomenon. Thoracic (A) and lumbar (B) MRI re-demonstrating compression fractures at T11, L1, and L2 and the dorsal extradural masses from T6 to T10.

In our emergency department, the patient reported increased lower extremity weakness and difficulty getting out of bed that had progressed over the past several weeks. On the morning of presentation, she acutely developed increased back pain, inability to ambulate, and loss of bowel and bladder function. She denied inciting trauma. On exam, the patient had significant weakness of bilateral lower extremities, consistent with an American Spinal Injury Association (ASIA) Impairment Scale of “B” [5]. Initial basic laboratory values were within normal limits other than the patient's baseline mild hematologic disturbances, which were unchanged from prior; hemoglobin was 9.6 g/dL with low mean corpuscular volume (MCV, 66.7 fL) and mean corpuscular hemoglobin concentration (MCHC, 31.6 g/dL), and red cell distribution width was elevated (RDW, 27.4%). MRI redemonstrated compression fractures at T11, L1, and L2 and the dorsal extradural masses but also revealed a new T9 compression fracture with vacuum phenomenon (Fig. 2). Given the patient's acute decline, she was taken to the operating room for urgent decompression and kyphoplasty.

**Fig. 3:**
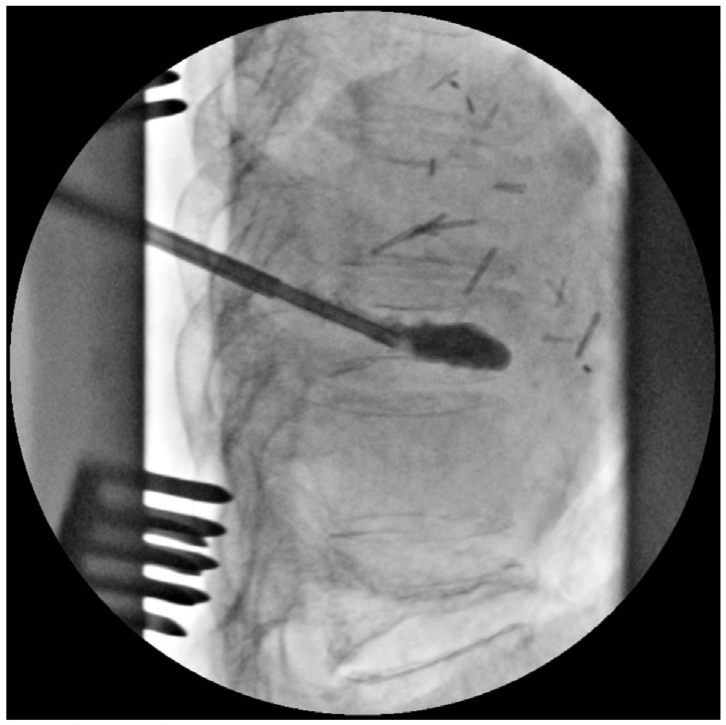
Intraoperative fluoroscopy confirming intravertebral containment of cement at T9.

The patient was placed in the prone position on a Jackson spine table. Baseline neuromonitoring signals were established with very poor potentials in the lower extremities, including only a small right foot response on motor evoked potentials, consistent with her baseline clinical exam. A midline incision was made and subperiosteal dissection was performed from T6 to T10. A Jamshidi needle was used to cannulate the left T9 pedicle under fluoroscopic guidance; a kyphoplasty balloon was inflated in the collapsed T9 body and cement was injected (Fig. 3). Kyphoplasty was performed prior to decompression to avoid passing the Jamshidi needle over the exposed dura at the decompression zone. Using a high-speed burr, troughs were created along the spinal laminar junction at each level, from T6 to T10. The laminectomy was performed using a lobster-tail technique. Red, rubbery tissue was encountered within the bone of the lamina and within the spinal canal, which was removed en bloc and sent as a pathology specimen. The spinal cord was examined and found to be decompressed, and neuromonitoring was stable from baseline. Due to the patient's history of multiple compression fractures, the patient would be at an elevated risk for proximal or distal junctional kyphosis following posterior fixation [6]. Thus, since no evidence of biomechanical instability observed, we did not pursue posterior fixation. The surgical site was thoroughly irrigated, a deep drain was placed, and the wound was closed in the usual layered fashion.

**Fig.4:**
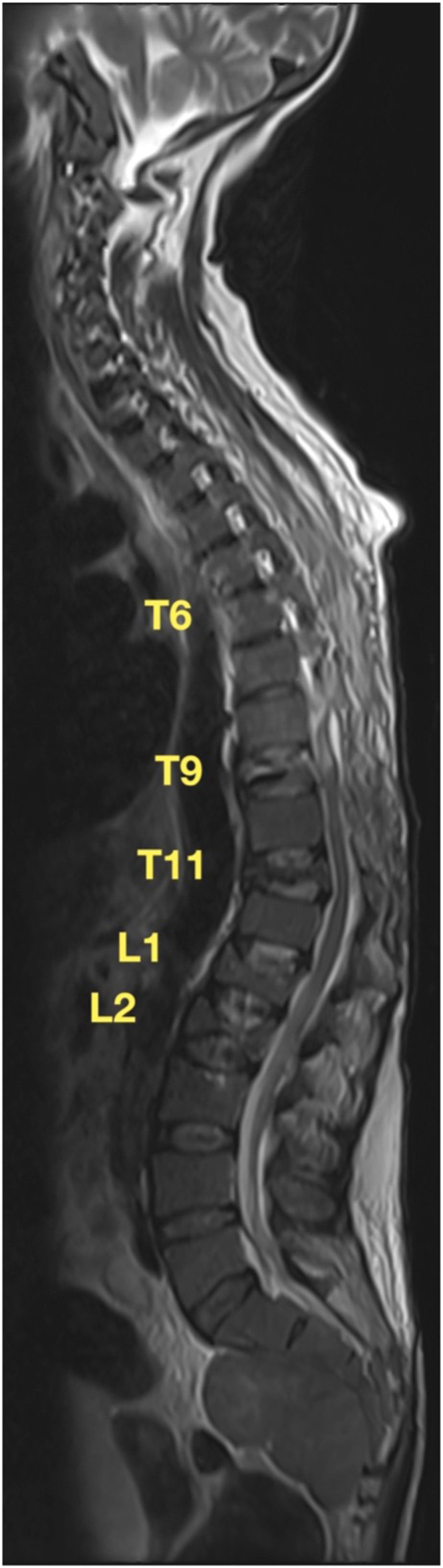
Immediate postoperative MRI demonstrating decompressed thoracic spinal cord with no residual dorsal extradural masses.

**Fig. 5:**
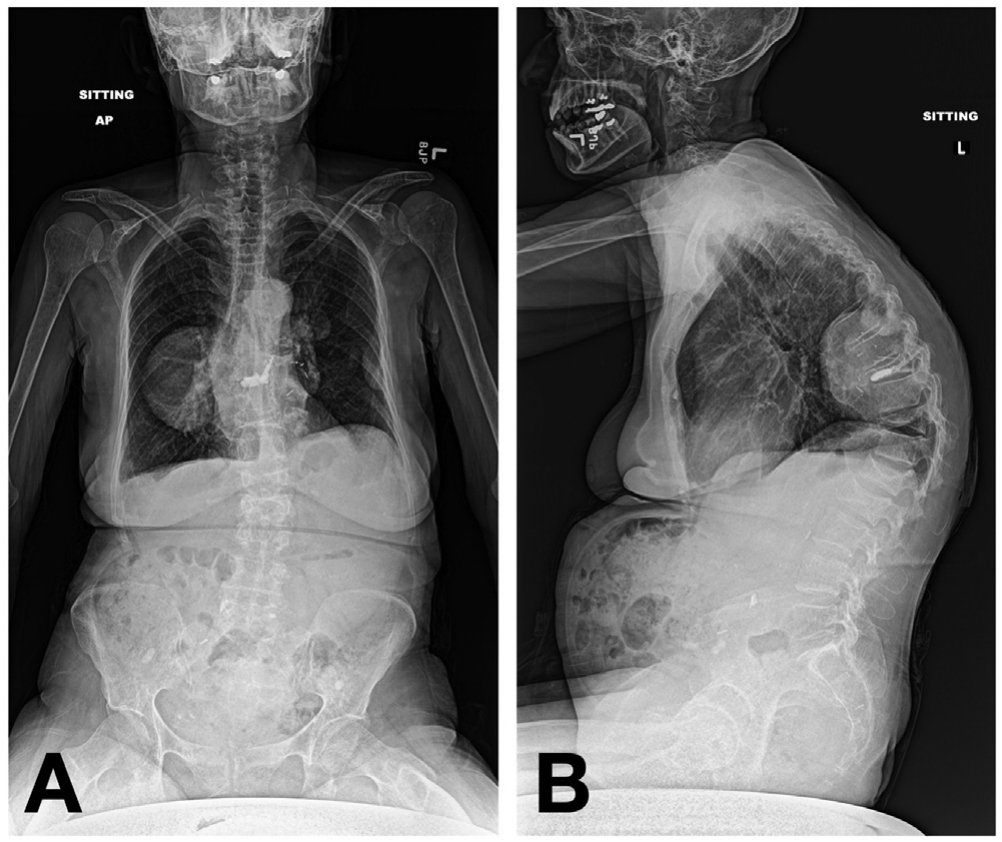
AP (A) and lateral (B) scoliosis x-rays at 3-week postoperative visit demonstrating maintained alignment of the spinal column with no cement extravasation or new loss of height.

**Fig. 6:**
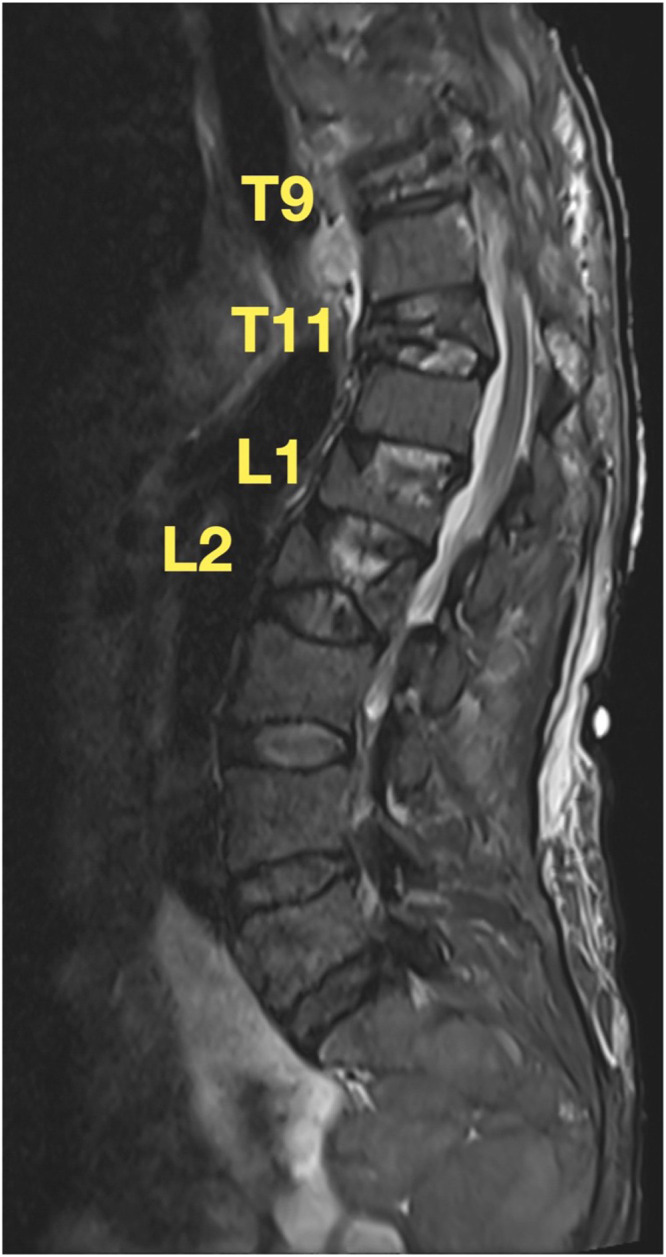
Lumbar MRI at 6-weeks postoperatively demonstrating postsurgical changes with no recurrence of dorsal extradural masses.

Postoperatively, the patient experienced immediate improvement in symptoms; her right lower extremity strength improved to baseline, and left lower extremity strength showed significant improvement (Fig. 4). On postoperative day 1, the patient's hemoglobin dropped to 7.4 g/dL; she was given 2 units of packed red blood cells which resulted in a sustained hemoglobin >10 g/dL throughout the remainder of her admission. The pathology was reported as prominent trilineage hematopoiesis and marked erythroid hyperplasia consistent with extramedullary hematopoiesis in context of thalassemia. The patient was discharged from the hospital to a skilled nursing facility for rehabilitation and physical therapy on postoperative day 12. At her first outpatient visit 3 weeks postoperatively, x-rays demonstrated maintenance of spinal alignment with no extravasation of cement or new loss of height (Fig. 5). She was noted to have superficial wound dehiscence distally and was prescribed oral antibiotics. At repeat wound check the following week, the patient was found to have increased dehiscence and drainage, at which point she was admitted to the hospital. MRI revealed no drainable fluid collection nor recurrence of her thoracic spine EMH masses (Fig. 6). She underwent posterior subfascial irrigation and debridement and was discharged back to rehab on antibiotics. At 3-month follow-up, the patient's wound was fully healed, and she was fully neurologically intact with normal sensation and 5 of 5 strength throughout.

## Discussion

There are several unique aspects of this case worthy of discussion. First, this patient had no focal neurological deficits at the time of her initial fall when she developed several acute thoracic and lumbar spine compression fractures; imaging at that time incidentally revealed the thoracic spine EMH. Over the next several months, her neurological deficits worsened and culminated in severe leg weakness, an acute inability to ambulate, a sudden loss of bowel/bladder function, and severe back pain akin to an ASIA Impairment Scale B spinal cord injury. Of note, at the time of her acute spinal cord injury, there was no reported inciting trauma and no retropulsion of the new T9 compression fracture. Thus, the major contributing factor to her spinal cord compression was likely the mass effect of the T-spine EMH masses. This timeline suggests that asymptomatic patients with thoracic spine EMH can develop progressive neurologic deterioration, culminating in acute spinal cord injury. While conservative management from a surgical perspective, as in this case, may be appropriate, patients should receive medical management for their underlying hematologic disorder to reduce the size and burden of the EMH masses and prevent the risk of symptomatic mass effect.

Indeed, our patient was followed closely by hematology before her acute neurologic change. Her thalassemia was managed with blood transfusions and deferoxamine transfusions to prevent iron overload. However, given her comorbid hemochromatosis, our patient could not receive regular transfusions, which may explain her EMH progression despite medical treatment. There are numerous treatment options and adjuncts available for patients with thalassemia and thoracic spine EMH, including hyper-transfusion, hydroxyurea, radiotherapy, surgical decompression, and most recently Luspatercept [2,7]. Luspatercept could have been a powerful adjunct in our patient's treatment since it improves hemoglobin levels and reduces transfusion dependence [7]. All therapies are effective individually and in combination, but due to the rarity of paraspinal EMH, there are no consensus treatment guidelines or recommendations [2,8]. These treatments are not without risk, and therapy must be directed on a case-by-case basis. Although surgery provides immediate relief of cord compression, removing the highly vascular mass can lead to bleeding and increases the risk of hemodynamic instability, which is particularly concerning in patients with baseline anemia [9]. Additionally, sudden resection of the compensatory hematopoietic tissue can worsen postoperative anemia [10]. In all patients with known thoracic spine EMH, however, surgery must always be considered in cases of severe neurologic deterioration, especially when there is concomitant instability akin to a severe spinal cord injury. In this case, the combination of multilevel decompression and single level kyphoplasty led to a full neurologic recovery in the short-term postoperative period.

Second, compression fractures in the setting of thoracic spine EMH appear to be rare. To our knowledge, there is only one reported case of a T11 compression fracture without vacuum phenomenon in the setting of thoracic spine EMH causing paraplegia; the patient was treated with T8 to L2 posterior decompression and fusion resulting in full neurologic recovery [11]. There are no reported cases of concomitant intravertebral vacuum phenomenon. Intravertebral vacuum phenomenon is a known sequela of compression fracture, occurring in up to 20% of osteoporotic fractures [12]. The presence of gas within the fracture reportedly diminishes the potential for proper healing, which may have led to progression of our patient's paravertebral masses of EMH [13]. As the initial damage and slowing of healing may have allowed for continuous extrusion of marrow through the bony defect and local release of hematopoietic stem cells, inducing the progression of the pre-existing masses. Though the theory that marrow extrusion from pathologic fractures leads to EMH has yet to be studied, previous case reports have documented a correlation between pathologic fractures and EMH. These reports describe EMH in patients without known marrow disease or myeloproliferative disorders but with pathologic fractures, with 1 report detailing a vertebral compression fracture [[14], [15]–16].

In conclusion, paraspinal EMH is a rare phenomenon that can occur in a number of hematologic disorders. Although many patients are asymptomatic and the disease is frequently diagnosed incidentally, it is important to consider addressing a patient's underlying hematologic disorder as this may help decrease tissue burden and reduce the risk of cord compression and neurologic deterioration. As demonstrated here, if untreated, asymptomatic patients can acutely progress to devastating neurologic deterioration, especially in the setting of additional spinal pathologies, such as concomitant vertebral body compression fractures. Urgent surgical decompression must always remain in consideration when treating patients with thoracic spine EMH as it offers the potential for a complete relief of symptoms and full neurologic recovery.

## Declaration of Competing Interest

One or more authors declare potential competing financial interests or personal relationships as specified on required ICMJE-NASSJ Disclosure Forms.
